# Neurological Disorders Related Neuronal Network Impairment: Function and Mechanism

**DOI:** 10.1155/2014/945078

**Published:** 2014-07-23

**Authors:** Sheng-tian Li, Yun Wang, Masayuki Matsushita

**Affiliations:** ^1^Bio-X Institutes, Shanghai Jiao Tong University, Shanghai 200240, China; ^2^Institutes of Brain Science and State Key Laboratory of Medical Neurobiology, Fudan University, Shanghai 200032, China; ^3^University of the Ryukyus, 207 Uehara, Okinawa 903-0215, Japan

Structural and functional neuronal networks provide the physiological basis for information processing and mental representations. Complex neurological disorders are characterized by structural and functional abnormalities in multiple brain areas involving several distinct brain systems. Recent advances in biology and medicine point to altered brain connectivity as a key feature of their pathophysiology. Knowledge and understanding of these deficits of neuronal networks have led to the development of animal models, successful therapies, and novel tools to characterize these clinical conditions and provide better care to patients. Inspired by the marked rise of scholarly research in these areas, this special issue attempts to provide timely collection of articles on neurological disorders, starting with recent findings on various deficits of neuronal networks and related signaling pathways in neurological disorders, and sheds light on the existing and emerging treatment concepts. The selected papers not only provide an overview of current open issues, but also identify potential lines for further research in the area of neurological disorders related neuronal network functions.

Epilepsy, particularly the temple lobe epilepsy (TLE), is a common neurological disorder worldwide; however, the underlying mechanism is still unknown. TLE is a most severe type of the epilepsy and with a high percentage becomes the untreatable seizure in clinic. In the current issue, we have three articles focused on the epilepsy study, in terms of the seizure origination in the neuronal network and functional abnormalities after the seizure. The papers by Y.-H. Li and colleagues and Y.-J. Shi and colleagues by using electrophysiological recording and mathematic modeling studied the brain structure relationship in generating the epileptiform activities during seizure initiation. They provide evidence that, in the seizure initiation period, the epileptiform activities are tended to spread from the thalamus to the hippocampus; in addition, the entorhinal cortex is not only a relay structure for epileptiform activities to the hippocampus, but also a more important brain structure in generating ictal discharges than hippocampus itself in generating epileptic seizure. Paper by S. Kong et al. further addresses the role of GABAergic inhibitory system in the chronic phase of the seizure. The paper described the phenomenon that, after a long silent period for seizure, the GABA synthetase, GABA transporters, and the GABA receptors are all significantly downregulated in the hippocampus, suggesting that the decreased GABAergic function directly contributes to the recurrent seizure occurrence.

Cognitive abilities rise steeply from infancy to young adulthood and then are either maintained or decline in older age. Brain activity studies have shown that healthy young adults develop better neurocognitive ability including working memory and attention. Evaluating working memory and attention in schizophrenia patients is usually based on traditional tasks and the interviewer's judgment. The article entitled “*A simple spatial working memory and attention test on paired symbols shows developmental deficits in schizophrenia patients*” by W. Song et al. reported a simple Spatial Working Memory and Attention Test on Paired Symbols (SWAPS). The SWAPS test has shown a plausible developmental pattern in healthy controls especially in difficulty load three and four, which developed well in the 20s group from the 10s group and then decline with the age increases.

In the spinal cord, the recurrent inhibitory circuit formed by Renshaw cells and motoneurons plays an important gated role in spinal motion loop. The dysfunction of Renshaw inhibition has been found to attribute to the cause of the motor neuron degeneration in various pathological conditions. The article entitled “*Reduced Renshaw recurrent inhibition after neonatal sciatic nerve crush in rats*” by L. Shu et al. is aimed to provide the relationships between peripheral nerve injury and spinal cord inhibitory circuit damage, particularly to the Renshaw recurrent inhibition pathway, during neonatal early development period, and discusses the possibility of Renshaw recurrent inhibition pathway being the target for neuroregeneration therapy.

Following stroke, a pathological neural plasticity termed postischemic long-term potentiation (i-LTP) often occurs over time, and emerging evidences from animal models suggest that such i-LTP plays important roles in ischemia. In their article “*Active calcium/calmodulin-dependent protein kinase II (CaMKII) regulates NMDA receptor mediated postischemic long-term potentiation (i-LTP) by promoting the interaction between CaMKII and NMDA receptors in ischemia,*” N. Wang et al. attempt to explore the mechanisms mediating i-LTP after stroke, especially in involvement of CaMKII activity and the enhancement of NMDA receptor mediated postsynaptic potentials.

The article entitled “*Enhanced expression of NR2B subunits of NMDA receptors in the inherited glaucomatous DBA/2J mouse retina,*” by L.-D. Dong et al. based on their observation of the relationships between increased expression of NMDA receptor subunit NR2B and the degeneration of retina ganglion cell in DBA/2J mice, the model for spontaneous secondary glaucoma proposed that progressive elevated intraocular pressure induced increase in NR2B expression may be associated with retina ganglion cells degeneration.

Vitamin B12 had been usually treated as sport nutrition, used to keep old people from getting anemic in past years. Recent studies have shown that vitamin B12 plays a key role in the normal functioning of the brain, nervous system, and the formation of blood. In their article entitled “*Methylcobalamin: a potential vitamin of pain killer*,” M. Zhang et al. summarized recent findings about the analgesic effects and mechanisms of methylcobalamin, an activated form of vitamin B12, in clinical low back pain, neck pain, and diabetic neuropathic pain patients.

Childhood emotional trauma contributes significantly to certain psychopathologies, such as posttraumatic stress disorder (PTSD). The article entitled “*The effects of early-life predator stress on anxiety- and depression-like behaviors of adult rats,*” contributed by L.-J. Chen et al., investigated the relationships between early-life predator stress and the anxiety- or depression-like behaviors in adulthood. They showed that, in both Wistar rats and the genetic depression model of WKY rats, the early-life predator stress did not increase anxiety- or depression-like behaviors in adulthood. They propose that early-life predator stress, at least for rats, does not induce PTSD.

The selected articles capture some of the state-of-the-art research issues in neurological disorders related signaling pathways and network functions and aid in expanding our understanding of disease mechanisms. The editors hope that this issue will serve to illustrate and help future researchers on this topic.

